# From DemenTia to DemenCia: Way More Than a Single Letter

**DOI:** 10.1093/geroni/igab046.1785

**Published:** 2021-12-17

**Authors:** Maggie Britton

**Affiliations:** University of Houston, Houston, Texas, United States

## Abstract

Hispanic or Latino/a/x/e (H/L) individuals are at 1.5x risk for Alzheimer’s Disease and Related Dementias (ADRD) compared to non-H/L White individuals. Although H/L individuals make up roughly 18% of the U.S. population, they are vastly underrepresented in ADRD research. For example, less than 9% of individuals in the National Alzheimer's Coordinating Center (NACC) data set are H/L. Collaborative efforts like the ECHAR Network are working to increase the representation of H/L individuals in ADRD research across the U.S. A non-exhaustive list of barriers to H/L participation include limited health literacy, perspectives on aging, and preferences for family-centered care that aligns with H/L cultural values (e.g., familismo). For example, H/L individuals are less likely to have a medical conceptualization of the Spanish translation for dementia. The cognate “demencia” is more likely to be conflated with alternative meanings like insanity, which may create barriers when developing community-facing recruitment and study materials.

